# Study on the Effect of Microstructure and Inclusions on Corrosion Resistance of Low-N 25Cr-Type Duplex Stainless Steel via Additive Manufacturing

**DOI:** 10.3390/ma17092068

**Published:** 2024-04-28

**Authors:** Yang Gu, Jiesheng Lv, Jianguo He, Zhigang Song, Changjun Wang, Han Feng, Xiaohan Wu

**Affiliations:** Research Institute of Special Steels, Central Iron & Steel Research Institute Co., Ltd., Beijing 100081, China; thiagoyoungkoo@163.com (Y.G.); lvllvlv@foxmail.com (J.L.); songzhigang@nercast.com (Z.S.); wangchangjun@nercast.com (C.W.); fenghan@nercast.com (H.F.); wuxiaohan@nercast.com (X.W.)

**Keywords:** additive manufacturing, duplex stainless steels, nano-inclusion, microstructure, corrosion resistance

## Abstract

Duplex stainless steels are widely used in many fields due to their excellent corrosion resistance and mechanical properties. However, it is a challenge to achieve duplex microstructure and excellent properties through additive manufacturing. In this work, a 0.09% N 25Cr-type duplex stainless steel was prepared by additive manufacturing (AM) and heat treatment, and its corrosion resistance was investigated. The results show that, compared with S32750 duplex stainless steel prepared by a conventional process, the combination value of film resistance and charge transfer resistance of AM duplex stainless steel was increased by 3.2–5.5 times and the pitting potential was increased by more than 100 mV. The disappearance of residual thermal stress and the reasonable distribution of Cr and N elements in the two phases are the reasons for the improvement of the corrosion resistance of AM duplex stainless steel after heat treatment. In addition, the extremely high purity of AM duplex stainless steel with no visible inclusions resulted in a higher corrosion resistance exhibited at lower pitting-resistance-equivalent number values.

## 1. Introduction

Duplex stainless steels (DSSs) are composed of both ferrite and austenite phases, combining the excellent mechanical properties of ferrite with the superior corrosion resistance of austenitic stainless steel [1,2,3]. They are widely used in industrial and maritime applications [4,5,6]. DSSs can achieve theoretical corrosion resistance performance and actual service performance higher than the 300 series unitary austenitic stainless steel at similar or lower raw material costs [7]. The corrosion resistance of DSSs mainly depends on composition and microstructure. 

AM technology is a production method that reduces costs and increases efficiency [8]. However, the relationship between the composition, process, microstructure, and properties of AM duplex stainless steel has not been systematically studied [9,10]. Research on the corrosion resistance of AM DSS steels are focused on utilizing the high forming temperature and rapid cooling rate characteristics of additive manufacturing to form numerous nano-sized oxide inclusions in the matrix [11,12]. Nano-inclusions bring benefits to the improvement of mechanical properties of duplex stainless steels, but the effect on the corrosion properties is not clear. Zhang et al. [13] investigated the substantial enhancement of mechanical properties in S32205 duplex stainless steel achieved through the incorporation of specialized nano-inclusions, with a powder oxygen content reaching 0.11 wt.%. Nano-inclusions bring benefits to the improvement of the mechanical properties of duplex stainless steels, but the effect on corrosion properties is not clear; while the measured corrosion potential increased, the pitting potential decreased. Haghdadi et al. [14] found that the corrosion resistance of AM 2205 duplex stainless steel was lower than that of hot rolled samples, and heat treatment was a necessary step to restore its pitting resistance; meanwhile, heat treatment was effective in restoring the duplex microstructure. Majld et al. [15] concluded that the decrease in the corrosion resistance of AM duplex stainless steel was caused by Cr_2_N precipitation. As reported by other investigations [16,17,18], the corrosion resistance of AM DSSs is commonly comparable or inferior to that of traditionally manufactured counterparts.

On this basis, this paper primarily focuses on a 25Cr-7Ni low-N (0.09 wt.%) duplex stainless steel through an established manufacturing process. Its corrosion resistance before and after heat treatment was investigated and compared, with the conventional S32750 duplex stainless steel used as a reference. Meanwhile, a variety of characterization methods for microstructure and substructure were used to analyze the reasons for the improvement of corrosion resistance from the perspective of inclusions, element distribution, and microstructure, which provided a research direction for breaking through the limitation of composition on the corrosion resistance of duplex stainless steel and achieving higher corrosion resistance under the premise of lower theoretical corrosion resistance.

## 2. Materials and Methods

**Figure 1:**
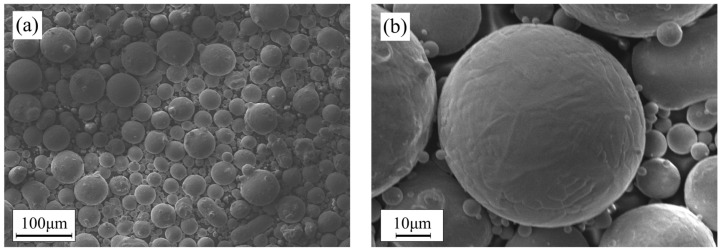
Powder SEM morphology: (**a**) 500× magnification; (**b**) 3000× magnification.

A 0.09% N 25Cr-type duplex stainless steel composition was designed. The low-nitrogen composition is designed to avoid the precipitation of various nitrides. Argon gas atomization was used to prepare 25Cr-type DSS powder, with a particle size distribution of 15–50 μm. The main chemical composition of the powder was measured via inductively coupled plasma atomic emission spectrometry (ICP–AES), and the results are shown in Table 1, with reference to the conventional preparation S32750 duplex stainless steel with a higher pitting-resistance-equivalent number (PREN) value, which is 42.1, while that of the 25Cr-type DSS is 38.6. Figure 1a shows the morphology observed under scanning electron microscopy (SEM) (supplied by FEI Co., Ltd., Hillsboro, OR, USA), revealing smooth, rounded particles without satellite powders. X-ray diffraction (XRD) analysis of the powder indicates a phase composition of 99.47% ferrite and 0.53% austenite, with no harmful phases.

The aforementioned powder was processed using a DLM-280 metal selective laser melting (SLM) machine (supplied by Pera Corporation Ltd., Shanghai, China). The building process took place on a 316 stainless steel substrate in a high-purity argon atmosphere (99.9%). The specific sintering parameters were as follows: laser input power (*P*) of 190 W, laser spot diameter of 0.1 mm, powder layer thickness (*h*) of 0.02 mm, line spacing (*t*) of 0.1 mm, scanning speed (*v*) of 850 mm/s, and a bidirectional scanning pattern with a 90° angle between each layer. The calculated energy density, obtained using Formula (1), was 117.65 J/mm³. The density of the as-built product, measured using the Archimedes drainage method, was 7.81 g/cm³, while the density of the conventional forged product was 7.82 g/cm³, resulting in a relative density of 99.87% [19].

(1) $$E=\frac{P}{v\cdot h\cdot t}$$

The heat treatment process was as follows: The AM samples were held at 1200 °C and 1100 °C for 1 h and then water-quenched. As a reference, the samples for the conventional process were held at 1100 °C for 1 h and then water-quenched.

The optical microscope (OM) specimens of 10 × 10 × 2 mm were mechanically ground and polished using a diamond polish with a granularity of 5 μm and then immersed in a potassium permanganate–sulfuric acid aqueous solution at 50 °C for 3 h. The OM microstructure was observed using a LEICA MEF4M optical microscope (supplied by Leica Microsystems Shanghai Ltd., Shanghai, China). Polished samples were subjected to statistical analysis for the macroscopic distribution and quantity of inclusions using the ASPEX metal inclusion analyzer (supplied by FEI Co. Ltd., Hillsboro, OR, USA), with a scanning area of 7 × 7 mm.

Transmission electron microscope (TEM) specimens were mechanically thinned to a thickness of 40 μm and then mechanically punched to obtain circular disks with a diameter of 3 mm. Further thinning of the disks was performed using a dual-jet electropolisher at 28 V and −20 °C. The electrolyte consisted of 10% perchloric acid and 90% anhydrous ethanol. Observation was carried out using a FEI TECNAI G2 F20 (supplied by FEI Co. Ltd., Hillsboro, OR, USA) operated at an acceleration voltage of 200 kV. Electron probe X-ray microanalyzer (EPMA) experiments were conducted using a JXA-8530F PLUS (Electronics companies of Japan, Tokyo, Japan) electronic probe. The backscattered electron diffraction (EBSD) experiments were conducted using a FEI Quanta650 field emission scanning electron microscope (supplied by FEI Co. Ltd., Hillsboro, OR, USA). EBSD samples were immersed in a 10% alcoholic hydrochloric acid solution and subjected to electrolytic polishing at a voltage of 25 V for 30 s. EBSD characterization was carried out using a FEI Quanta650 field emission scanning electron microscope, and the data were processed using Channel 5 software. The EBSD data collection was conducted with a step size of 0.6 μm and a resolution of 400 × 400.

The electrochemical experiments were carried out using a standard three-electrode system, with the polished sample to be tested as the working electrode (WE), the platinum electrode as the counter electrode (CE), and the saturated calomel electrode (SCE) as the reference electrode. The electrochemical testing employs a 3.5 wt.% sodium chloride solution. Prior to the test, cathodic polarization was performed at −1 V vs. Ref. for 3 min to remove the passivation layer on the sample surface. Subsequently, an open-circuit (OC) voltage test was conducted for half an hour, allowing the voltage to stabilize within the range of ±10 mV before proceeding to the next step. The frequency range for electrochemical impedance spectroscopy (EIS) was from 10^−2^ Hz to 10^5^ Hz. The impedance measurement signals had an amplitude of ±10 mV vs. OC sinusoidal waveforms, and the curve fitting was performed using ZSimpWin software (Version 3.6 EChem Software, Ann Arbor, MI, USA, http://www.echemsw.com (accessed on 2 March 2024)). For cyclic polarization curve testing, the initial voltage was set at −0.5 V vs. OC. The anodic current was scanned until it reached 100 μA, and then, a reverse scan was performed, terminating at the open-circuit potential. The scanning rate was 0.5 mV/s.

## 3. Results

### 3.1. Microstructure

**Figure 2:**
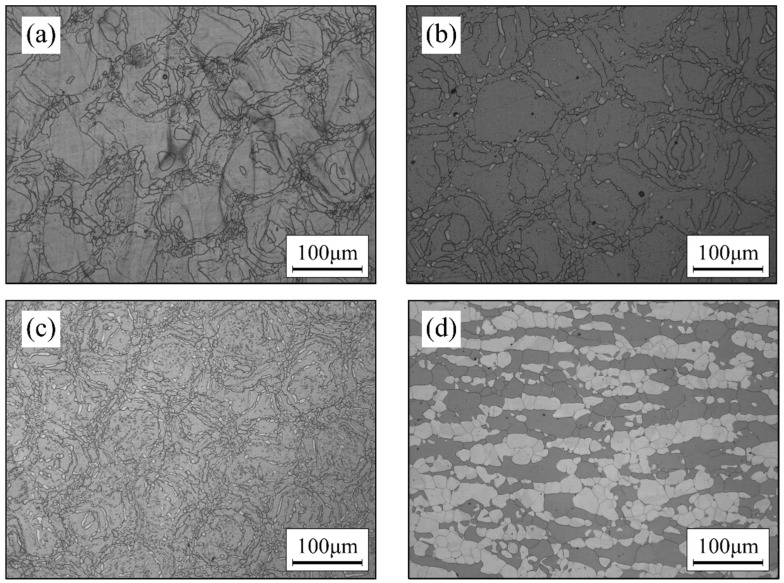
OM image of the specimen before and after heat treatment: (**a**) AM sample before heat treatment, (**b**) AM sample heat treated at 1200 °C for 1 h, (**c**) AM sample heat treated at 1100 °C for 1 h, and (**d**) conventionally processed sample.

In Figure 2, the OM microstructure of the test samples before and after heat treatment is presented. In Figure 2a, the microstructure of the AM sample appears as a mosaic-like structure, with the minimum unit size of the mosaic structure being approximately 100 × 100 μm. This structure consists of larger grains in the central region and finer, fragmented grains at the edges. The unique microstructure is primarily determined by the building process, where the laser diameter is 100 μm, and the scanning interval is also 100 μm. With each layer formed at a 90° angle to the previous one, the building strategies result in the mosaic-like microstructure. Additionally, previous studies have indicated that the untreated microstructure is a unitary ferrite structure [19].

Figure 2b and Figure 2c, respectively, illustrate the OM microstructure after heat treatment at 1200 °C and 1100 °C for 1 h. Compared to the untreated microstructure, new austenite precipitated along the ferrite grain boundaries and within the grains after heat treatment, transforming the microstructure from unitary ferrite to a dual-phase structure. It is noteworthy that the regular mosaic pattern at the macroscopic level remained intact. This is mainly attributed to the low-nitrogen (N) composition design. Even after thorough heat treatment (1 h), the newly formed austenite impeded the mutual merging of the original ferrite grains.

As a reference, the microstructure of the S32750 duplex stainless steel with standard composition after conventional hot rolling and solution treatment (1100 °C × 1 h) is shown in Figure 2d. It can be observed that the microstructure of the AM samples is significantly different from that of the duplex stainless steel produced through the conventional process. The main differences lie in the phase morphology and grain size. Conventional processes including casting, forging, hot rolling, and heat treating produced larger grain sizes compared to one-off rapid prototyping of AM samples. At the same time, the conventional process 25Cr duplex stainless steel has significantly more austenite due to its higher N content. In terms of phase distribution, the two phases of AM sample were mainly distributed along the build trajectory, while the two phases of the conventional processes were mainly distributed along the rolling direction. The differences in corrosion resistance performance resulting from structural variations between the two processes are discussed in detail in the following sections.

### 3.2. Corrosion Resistance

**Figure 3:**
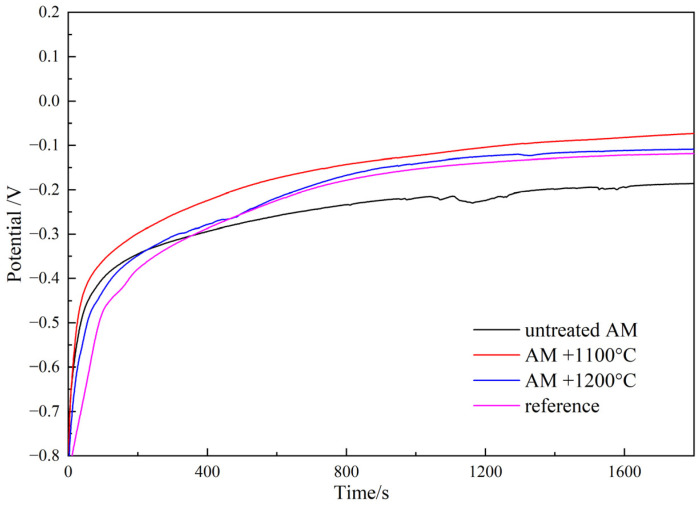
OCP of duplex stainless steels under different preparation processes.

The open-circuit potential (OCP) of the untreated AM sample was the lowest after solution treatment, which was slightly better than that of the conventional process. In Figure 3, OCP curves after cathodic polarization at −800 mV for 3 min are presented for several samples. After a test duration of 1800 s, the OCP values of all samples stabilized around −100 mV. The OCP value of the untreated AM sample was the most negative and was significantly lower than the other samples. After heat treatment, the OCP values noticeably increased and were all better than the conventional process samples [20]. A lower OCP value indicates the higher electrochemical activity of the tested sample. Therefore, the untreated AM sample exhibited the highest corrosion tendency and had the worst corrosion resistance, while after heat treatment, its corrosion resistance was better than that of the conventional process sample.

**Figure 4:**
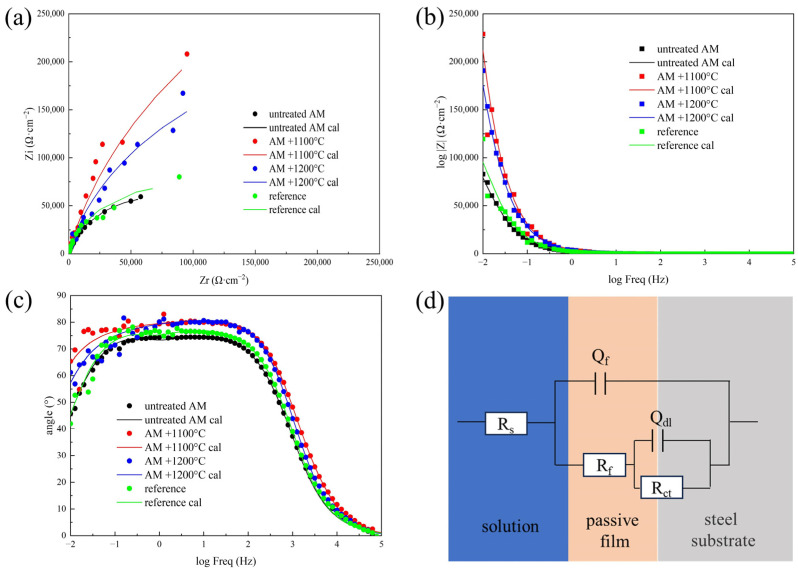
Impedance spectra of samples under different preparation processes: (**a**) Nyquist diagram; (**b**,**c**) Bode diagram; (**d**) equivalent circuit for fitting EIS diagram.

Figure 4 shows the electrochemical impedance spectra of different samples at open-circuit potential. In the Nyquist diagram, all capacitance reactance arcs formed incomplete semicircles, indicating that the corrosion mechanism of the tested steel in the 3.5% NaCl solution remains unchanged regardless of the preparation method. The capacitive reactance arcs of both the untreated AM sample and the sample from conventional processes were small and relatively closed to each other. However, after heat treatment, the capacitive reactance arc of the AM sample noticeably increased, with the most significant increase observed in the sample treated at 1100 °C. This phenomenon reflects the improved corrosion resistance of the AM sample after heat treatment. As can be seen from the Bode plot, the heat-treated AM sample had a higher impedance modulus (|*Z*|) at low frequencies, indicating excellent corrosion resistance. Similarly, the phase angle plot shows excellent passivation performance.

The impedance spectra data were fitted using the equivalent circuit shown in Figure 4d. R_s_ represents the electrolyte resistance, the constant phase element *Q*_f_ corresponds to the passive film capacitance, *R*_f_ is the passive film resistance, and *Q*_dl_ and *R*_ct_ represent the double-layer capacitance and charge transfer resistance, respectively [21,22]. The membrane values of different samples in the high-frequency region are closely related to the corrosion resistance of the passive film, while the low-frequency region may be associated with charge transfer resistance [20]. The fitting results using the aforementioned equivalent circuit are presented in Table 2. The combination of *R*_f_ and *R*_ct_ reflects the holistic corrosion resistance of the samples. From the table, it can be seen that the n values of *Q*_f_ and *Q*_dl_ are close to 1, indicating that the constant phase elements were close to pure capacitors. A greater value of n designates a decrease in surface inhomogeneity, associated with a strong adsorption of polymer and inhibitor [23]. This suggests effective passivation of the passive film. Comparing the values of *R*_f_ and *R*_ct_ for different samples, it was found that the charge transfer resistance *R*_ct_ was significantly larger than *R*_f_, especially for the heat-treated AM sample. This indicates that charge transfer was more difficult, reflecting the greater difficulty for electrons to escape from the surface of the substrate, making it more challenging for metal atoms on the substrate to be oxidized into ions. The passive layer on the stainless steel surface is composed of substances such as Cr_2_O_3_, Cr(OH)_3_, Fe_2_O_3_, Fe_3_O_4_, etc. [24,25]. Since there were fewer metal ions involved in the formation of these substances, the structure, thickness, and formation processes of the passive film were different from the other two samples, which is also the reason for the smaller R_f_ in the heat-treated AM sample. Although the resistance of electrons passing through the passive film was relatively small, the n values of *Q*_f_ for all the heat-treated AM samples were 1, indicating that the capacitive properties of the passivation film of the samples are more pronounced, and the hindering effect on the ions is more obvious.

In summary, the corrosion resistance of the test steel can be impacted by the magnitude of *R*_f_ + *R*_ct_ value (the combination value of film resistance and charge transfer resistance). It can be seen that the corrosion resistance of the untreated AM sample was the worst, and it was significantly improved after heat treatment. The *R*_f_ + *R*_ct_ value of the AM sample after heat treatment at 1200 °C is 3.2 times of that of the conventional processes sample, and it increased to 5.5 times at 1100 °C, which are both much better than that of the conventional sample.

**Figure 5:**
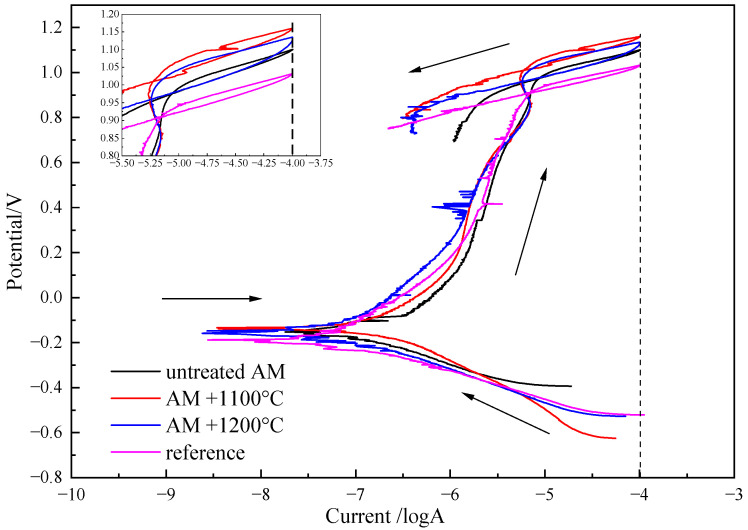
Potentiodynamic polarization curve of duplex stainless steels under different preparation processes.

Figure 5 presents the cyclic polarization curves of different samples in a 3.5% NaCl solution, showing a similar overall trend and indicating a common corrosion mechanism. Combining the curves, it is evident that the anodic curves of different samples exhibited clear passivation behavior. Through the analysis of the polarization curves, information such as the corrosion current density (*I*_corr_), critical pitting potential (*E*_p_), and corrosion potential (*E*_corr_) can be obtained. When the applied current exceeded the passivation region, the current density suddenly increased, and pitting corrosion was considered to occur when the current density reached 100 μA/cm². The potential corresponding to this point is defined as *E*_p_. The stability of the passive film is characterized by the difference between *E*_p_ and the corrosion potential *E*_corr_. When the potential reached *E*_p_, the curve began to reverse scan, and during the reverse scan, the current exhibited a hysteresis phenomenon. The intersection of the reverse scan curve with the forward scan anode curve is repassivation potential, defined as *E*_r_. The difference between *E*_p_ and *E*_r_ is used to characterize the repairability of the passive film on the sample, i.e., the repassivation performance.

From Table 3, it can be observed that the conventional process sample had the most negative corrosion potential, followed by the untreated AM sample. After heat treatment, the corrosion potential of the AM sample was improved, especially after the 1100 °C heat treatment, where the corrosion potential was significantly higher than other samples, indicating the least tendency for corrosion to occur. The pitting potential followed the same pattern as the corrosion potential. After heat treatment at 1100 °C and 1200 °C, the pitting potential of AM samples was 128.5 mV and 103.4 mV higher than that of conventional samples, respectively. In addition, the *E*_p_-*E*_corr_ values of all AM samples were larger than that of the conventional sample, implying that the AM samples had a larger passivation interval. In contrast to the pattern of pitting potential, the conventional process sample exhibited the best repassivation performance, while the heat-treated AM samples showed relatively poor repairability of the passive film, with a larger *E*_p_-*E*_r_ value. In summary, the AM samples after heat treatment have a lesser corrosion tendency, a higher pitting potential, and a larger passivation interval, although the repassivation performance is slightly worse; overall, the corrosion resistance is better than that of conventional process samples.

## 4. Discussion

In the above results, it is evident that the AM samples after heat treatment, despite having initially lower PREN values and higher oxygen content, exhibited better corrosion resistance performance than the conventional processed samples. This section discussed the reasons for the superior corrosion resistance performance of the AM samples and the variations in the corrosion resistance performance of AM samples under different conditions. In this section, the mechanism of corrosion resistance enhancement of AM samples is discussed in terms of phase composition, elemental distribution, substructure, and inclusions.

### 4.1. Influence of Phase Composition and Substructure on Corrosion Resistance Properties

For DSSs, the coexistence of two phases with a reasonable distribution of elements is conducive to the improvement of corrosion resistance performance [26]. It is generally considered that the corrosion resistance of austenite is superior to that of ferrite in dual-phase stainless steel [27]. Within the compositional range of dual-phase stainless steel, unitary ferritic structure without heat treatment is also unfavorable to corrosion resistance from an element distribution perspective.

**Figure 6:**
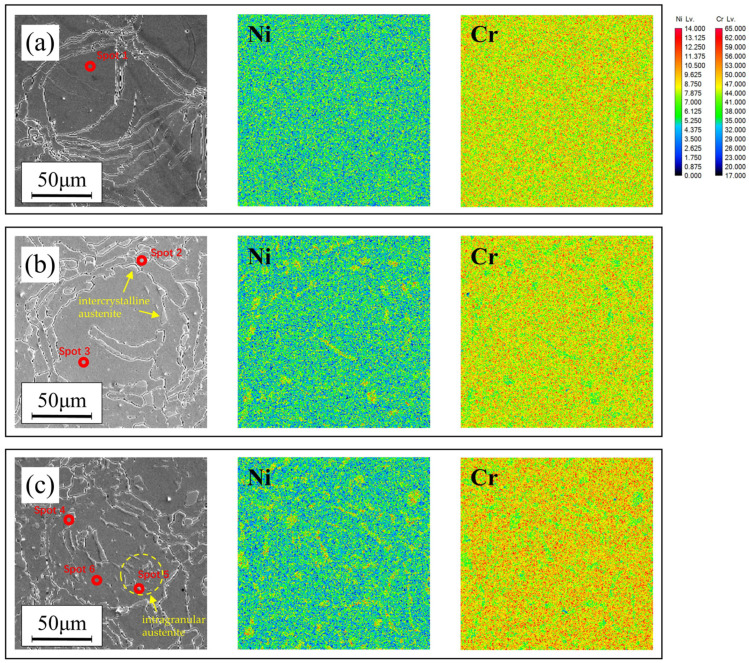
EPMA mapping results of AM samples of different processes: (**a**) untreated, (**b**) 1200 °C solid solution, (**c**) 1100 °C solid solution.

Through OM microstructure analysis, the variation in austenite content was examined: 0% for untreated samples, 8.2% for heat-treated samples at 1200 °C, and 16.3% for heat-treated samples at 1100 °C. Under the same composition, the change in the proportion of the two phases resulting in the variation of element distribution is the main cause of the change in corrosion resistance. Figure 6 presents the EPMA mapping results of additive manufacturing samples at different heat treatment temperatures. It can be observed that after heat treatment at 1200 °C, austenite appeared in the intercrystalline regions of the AM samples, with Ni elements enriched in austenite and Cr elements enriched in ferrite [28]. After heat treatment at 1100 °C, austenite content increased, with austenite appearing not only in the intercrystalline regions but also within the grains. Ni was highly enriched in both intercrystalline and intragranular austenite. Compared to heat treatment at 1200 °C, the enrichment of Cr elements in the ferrite phase was more pronounced at this temperature. The appearance of austenite led to a more balanced distribution of elements between the two phases, which was beneficial for improving corrosion resistance performance.

Furthermore, the intercrystalline regions had higher energy, providing a pathway for rapid diffusion of elements. This resulted in compositional differences between intergranular and intragranular austenite. EPMA spot scanning was conducted to characterize the elements content of different phase in AM samples under different conditions, with results shown in Table 4. For DSSs, corrosion resistance primarily depends on the content of Cr, Mo, and N elements. The N element content is mainly enriched in the austenite phase, while Cr and Mo have higher concentrations in the ferrite phase [29]. Typically, the impact of N element on corrosion resistance performance is greater than that of the other two elements. Additionally, the austenite phase exhibits a more corrosion-resistant microstructure, while ferrite often becomes the weaker phase during corrosion processes [30]. Table 4 shows that the Cr and N contents in intragranular austenite were both higher than those in intercrystalline austenite, making it the most corrosion-resistant component among intragranular austenite, intercrystalline austenite, and ferrite. Furthermore, the appearance of intragranular austenite led to further enrichment of Cr elements in the ferrite, thereby enhancing the corrosion resistance of the weakest phase (ferrite). In summary, the combination of intragranular and intercrystalline austenite phases with ferrite ensures a more rational distribution of elements, thereby improving the corrosion resistance performance.

For AM samples, the difference in corrosion resistance between the samples before and after heat treatment is not only due to the ratio of the two phases, the morphology of the two phases, and the elemental distribution between the two phases but is also affected by the number of grains and substructures [31,32,33]. During the building forming process, the central part of the laser had higher energy, allowing grains to grow sufficiently, while the edges, due to the reduction in laser energy, have relatively smaller grain sizes. Smaller grain size implies more grain boundaries, substructures, and dislocation density.

**Figure 7:**
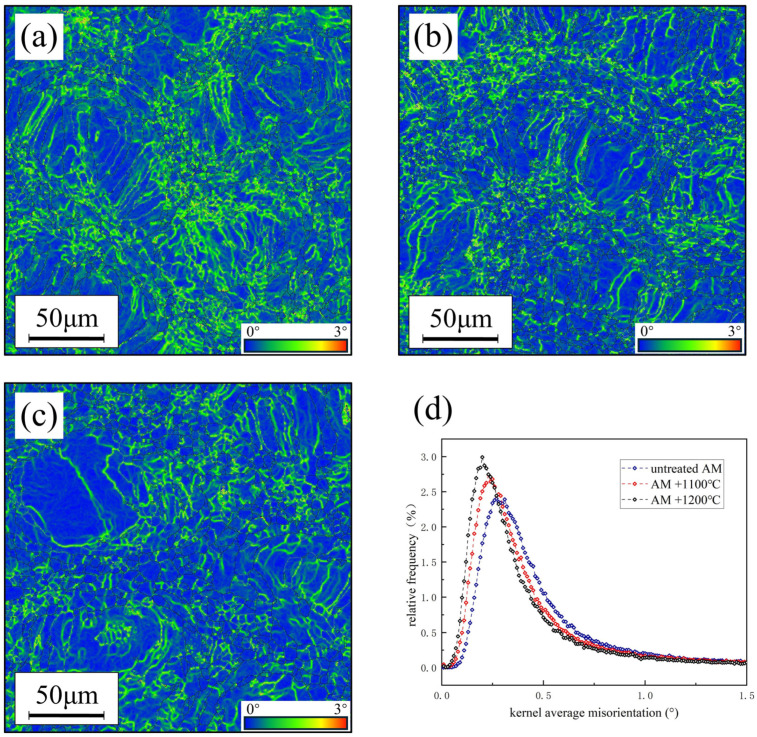
EBSD KAM test results of AM sample: (**a**) untreated, (**b**) 1200 °C solid solution, (**c**) 1100 °C solid solution, and (**d**) KAM distribution.

Figure 7 illustrates the EBSD kernel average misorientation (KAM) test results for AM specimens in different states. KAM can be used to characterize the stress state and deformation extent [11,34]. None of the AM samples used for testing in this work were plastically deformed, so a higher KAM indicates a higher stress state. Therefore, it is inferred that the high KAM originates from the high dislocation density. The sources of these dislocations were mainly thermal stress residues and supersaturated solid solutions of elements during rapid cooling. After heat treatment, thermal stresses were removed, elements were redistributed between the two phases, the peak of the KAM value was shifted to the left, the peak width decreased, and the dislocation density decreased.

**Figure 8:**
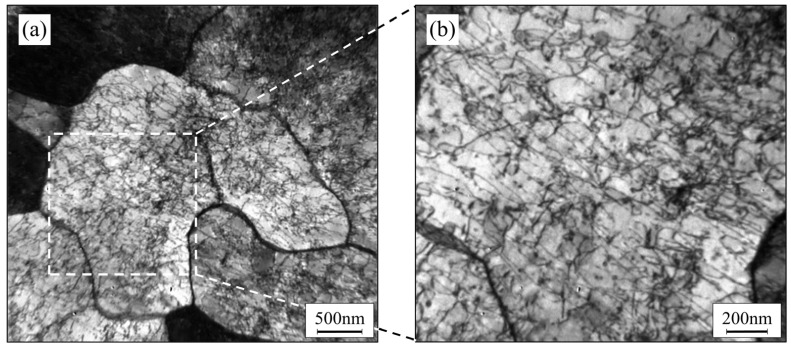
TEM morphology of dislocations in untreated AM sample (**a**) and selected area enlargement (**b**).

The untreated AM samples exhibited a unitary ferritic structure. However, at room temperature, the saturation N element solubility in ferrite is only 0.07% [35], while the N content of the AM sample was 0.09%, which was entirely oversaturated and dissolved in the ferrite. Figure 8 shows TEM images of the untreated samples, revealing that even without deformation, the ferrite has an extremely high dislocation density due to the oversaturation of N elements and residual thermal stress from the rapid cooling during the forming process. This is one of the reasons why the corrosion resistance performance of untreated samples was lower than that of heat-treated samples.

### 4.2. Effect of Inclusions on Corrosion Resistance

There are various factors influencing the pitting corrosion resistance of stainless steel. For conventional process stainless steel, inclusions are one of the significant contributors to the deterioration of its corrosion resistance. During the conventional smelting process, unavoidable steps like deoxidation introduce inclusions such as Al_2_O_3_, MnO, and others [36,37]. These inclusions have higher melting points than the alloy, and their stable nature makes it challenging to eliminate them through subsequent processing steps. These inclusions are not only difficult to eliminate but also have relatively large sizes, leading to a certain degradation in the final mechanical and corrosion resistance properties of stainless steel. Therefore, the modification of inclusions and the enhancement of the purity of stainless steel are crucial measures for optimizing its corrosion resistance performance.

**Figure 9:**
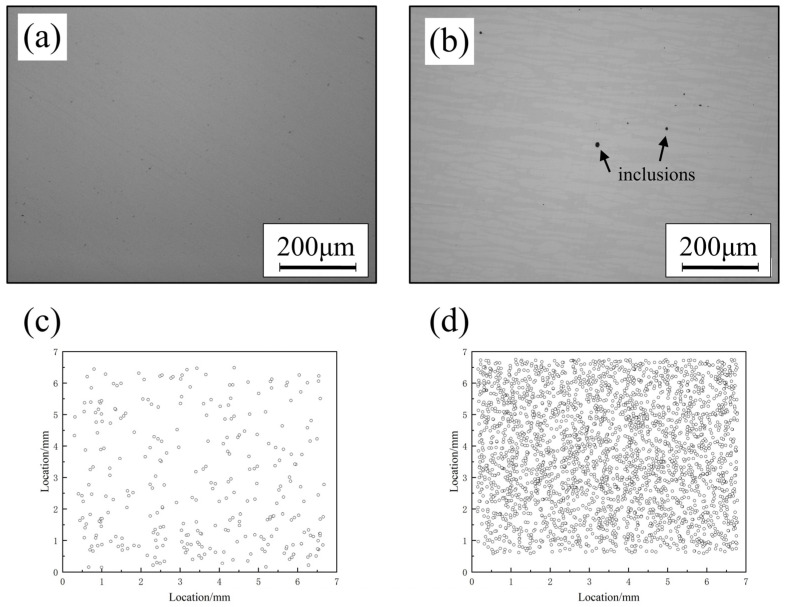
Distribution of inclusions: (**a**) AM sample surface morphology (OM), (**b**) conventionally processed surface morphology (OM), (**c**) AM sample inclusion distribution (ASPEX), and (**d**) conventional processed sample inclusion distribution (ASPEX).

Figure 9a,b, respectively, show surface micrographs of the polished states of the AM samples and the conventionally processed samples. It can be observed that the AM samples exhibited extremely high purity, with virtually no visible inclusions. In contrast, the conventionally processed sample showed a significant presence of black inclusions. Furthermore, the distribution of inclusions was statistically analyzed using ASPEX inclusion analysis, with the results depicted in Figure 9c,d. It should be noted that the substances detected by ASPEX are still customarily referred to as inclusions, even though they are much smaller than common inclusions and are thus unobservable under a metallurgical microscope [14]. In the untreated AM samples, a minimal number of inclusions was observed. Conversely, the conventional process samples exhibited a significantly higher number of inclusions, which became one of the significant factors deteriorating its corrosion resistance performance. This is why, despite having a higher PREN value, the corrosion resistance performance of the conventional processed samples was weaker than that of the AM samples.

**Figure 10:**
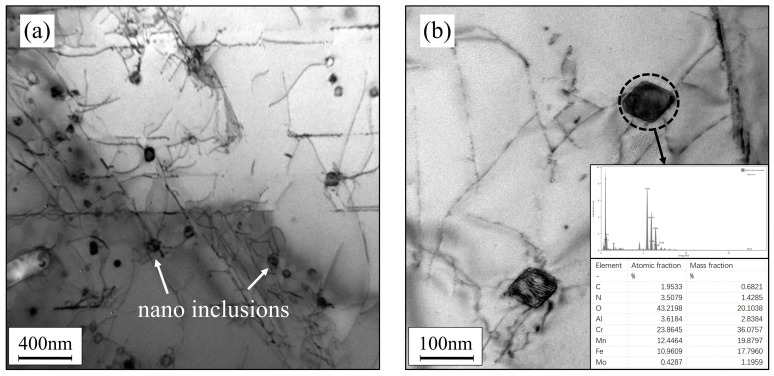
TEM morphology of inclusions in heat-treated AM samples: (**a**) 25,000× magnification; (**b**) 100,000× magnification.

However, it is worth noting that the oxygen (O) element content in the AM samples was as high as 0.02%, much higher than the oxygen content in the conventionally processed samples (0.002%). Interestingly, O, as the primary forming element of inclusions, did not appear in the form of large-sized inclusions in the AM samples. To investigate this, we explored the presence of oxygen in the AM samples. Figure 10 presents the TEM microstructure of the AM samples, revealing numerous particles with sizes ranging from 20 to 100 nm. STEM characterization of their elemental composition indicated the presence of particles rich in oxygen, manganese, and chromium. The formation of these nanoscale oxides was primarily attributed to the unique process of AM.

Through the powder bed fusion process, high-purity powder without inclusions could be obtained. However, the powder-making process unavoidably introduced oxygen, leading to a higher oxygen content. In the additive manufacturing process, the laser created a high-temperature melt pool on the powder bed, where numerous oxides rapidly formed. Due to the relatively fast scanning speed (850 mm/s) and the short duration of the melt pool, coupled with the extremely rapid cooling rate (10^5^–10^7^ K/s) [35], these oxides did not have enough time to grow into large inclusions. Instead, they formed nanoscale inclusions. The nanoscale inclusions significantly enhanced the macroscopic purity compared to conventional processes, which ultimately resulted in better corrosion resistance performance at a lower PREN in the AM samples.

## 5. Conclusions

A 0.09% N 25Cr-type duplex stainless steel was prepared by AM and heat treatment, and its corrosion resistance was investigated. The results indicate that the refinement of inclusion size has a favorable impact on its corrosion resistance performance. With a lower PREN value, additive manufacturing products can achieve superior actual corrosion resistance performance.

(1) The corrosion resistance of 0.09% N 25Cr-type AM test steel after heat treatment is superior to that of S32750 duplex stainless steel with the same Cr content when hot rolled and in solid solution condition. In a 3.5% wt.% NaCl solution, the combination value of film resistance and charge transfer resistance of AM samples in solid solution at 1100 °C and 1200 °C was 5.5 times and 3.2 times that of conventional S32750 duplex stainless steel, respectively; the pitting potential was 128.5 mV and 103.4 mV higher, respectively;
(2) The disappearance of residual thermal stress and the reasonable distribution of Cr and N elements in the two phases are the reasons for the improvement of the corrosion resistance of AM duplex stainless steel after heat treatment. When the heat treatment temperature decreased from 1200 °C to 1100 °C, the austenite phase morphology changed from intercrystalline austenite to intercrystalline and intragranular austenite. The Cr and N contents in intragranular austenite were both higher than those in the intercrystalline austenite, also leading to further enrichment of Cr elements in the ferrite and enhancing the corrosion resistance of AM DSSs;
(3) The influence of inclusions on corrosion resistance of DSSs is more obvious than PREN value. Unlike the large particle inclusions commonly found in duplex stainless steel prepared by conventional processes, the oxygen in AM samples only exists in nanoscale Mn and Cr oxides due to the particularity of AM technology. The extremely high purity of AM duplex stainless steel with no visible inclusions resulted in a higher corrosion resistance exhibited at lower PREN values.

## Figures and Tables

**Table 1:** Chemical composition of the tested DSSs.

	Cr	Ni	Mo	N	Mn	O	Si	C	PREN
AM powder	24.70	6.52	3.74	0.098	0.55	0.028	0.35	0.0050	38.6
S32750	25.39	6.72	3.71	0.28	0.40	0.0022	0.43	0.020	42.1

Note: PREN = *W*_Cr_ + 3.3 × *W*_Mo_ + 16 × *W*_N._

**Table 2:** Fitted electrochemical parameters for EIS data of duplex stainless steels under different preparation processes.

	*R*_s_ (Ω cm^2^)	*Q*_f_ (μF/cm^2^)		*R*_f_ (Ωcm^2^)	*Q*_dl_ (μF/cm^2^)		*R*_ct_ (Ωcm^2^)	*R*_f_ + *R*_ct_ (Ωcm^2^)
		Y_0_	n		Y_0_	n		
Untreated AM	6.53	91.97	0.855	1.82 × 10^4^	12.25	0.978	1.27 × 10^5^	1.46 × 10^5^
AM + 1100 °C	5.56	13.82	1.000	7.85	34.82	0.827	9.31 × 10^5^	9.31 × 10^5^
AM + 1200 °C	6.16	17.95	1.000	11.12	35.7	0.805	5.22 × 10^5^	5.22 × 10^5^
Reference	6.06	80.08	0.874	2.76 × 10^4^	9.959	1.000	1.41 × 10^5^	1.68 × 10^5^

**Table 3:** Electrochemical parameters for polarization curve of duplex stainless steels under different preparation processes.

	*E*_corr_/mV	*I*_corr_/μA	*E*_p_/mV	*E*_p_-*E*_corr_/mV	*E*_r_/mV	*E*_p_-*E*_r_/mV
Untreated AM	−153.46	0.282	1100.75	1254.21	972.84	127.91
AM + 1100 °C	−134.99	0.293	1160.33	1295.32	1008.97	151.36
AM + 1200 °C	−142.91	0.265	1135.19	1278.10	985.91	149.28
Reference	−189.34	0.272	1031.80	1221.14	909.27	122.53

**Table 4:** EPMA spot scanning results.

Spot	Phase	The Element Content (wt.%)						
		Ni	Cr	Mo	Mn	C	N	Fe
1	Ferrite	6.63	25.30	3.65	0.48	0.64	0.15	Bal.
2	Intercrystalline austenite	8.87	22.33	2.74	0.41	0.43	0.61	Bal.
3	Ferrite	6.32	25.69	3.67	0.43	0.60	0.05	Bal.
4	Intercrystalline austenite	8.33	22.59	2.81	0.64	0.54	0.36	Bal.
5	Intragranular austenite	7.00	24.65	3.25	0.75	0.51	0.41	Bal.
6	Ferrite	6.07	25.99	3.72	0.52	0.59	0.06	Bal.

## Data Availability

Data are contained within the article.
